# EHMTI-0157. Specific strength training compared with interdisciplinary counselling for girls with tension-type headache – a randomised controlled trial

**DOI:** 10.1186/1129-2377-15-S1-E37

**Published:** 2014-09-18

**Authors:** B Tornøe, LL Andersen, JH Skotte, R Jensen, C Jensen, BK Madsen, G Gard, L Skov, I Hallström

**Affiliations:** 1 Pediatric department, Herlev Hospital, Copenhagen, Denmark; 2 Working environment, National Research Centre for the Working Environment, Copenhagen, Denmark; 3 Department of Neurology Glostrup hospital University of Copenhagen, Danish Headache Centre, Copenhagen, Denmark; 4 Aps, Huge Consulting, 4690 Haslev, Denmark; 5 Faculty of Medicine, Department of Health Sciences, Lund, Sweden; 6 Pediatric department, Herlev Hospital University of Copenhagen, Herlev, Denmark

## Background

Childhood tension-type headache (TTH) is a prevalent and debilitating condition for the child and family. Low-cost non-pharmacological treatments are usually the first choice of professionals and parents. The study examined the outcomes of specific strength training for girls with TTH.

## Methods

Forty-nine girls 9-18 years with TTH were randomised to patient education programmes with 10 weeks of strength training compared with counselling by nurse and physiotherapist. Primary outcomes were headache frequency, intensity and duration; secondary were neck-shoulder muscle strength, aerobic power and pericranial tenderness, measured baseline, after intervention and at 12 weeks follow-up. HRQOL questionnaires were assessed at baseline.

## Results

For both groups headache frequency decreased significantly, p=0.001 and likewise for duration p=0.022, with no significant between-group differences. The odds of having headache a random day decreased during 22 weeks by 0.65 (0.50-0.84) (OR (95%CI)). For both groups neck extension strength decreased significantly with a decrease in cervicothoracic ratio to 1.7, indicating a change in muscle balance. In the training group shoulder strength increased ≥ 10 % in 5/20 girls and estimated VO2 max increased ≥ 15% for 4/20 girls.

## Conclusions

Both training and counselling lead to headache reduction. Adjusting muscle-balance seems to precede strength gains. Exercising might lead to important changes in the child’s physical capability and health. Restructuring patient education and examining dose-response of exercising is recommended.

No conflict of interest.

